# (1*S*,4*S*,5*S*,6*R*)-6-(4-Bromo­phen­yl)-5-nitro­bicyclo­[2.2.2]octan-2-one

**DOI:** 10.1107/S1600536809001275

**Published:** 2009-01-17

**Authors:** Aibao Xia, Jie Tang, Yifeng Wang, Junrong Jiang, Shuping Luo

**Affiliations:** a State Key Laboratory Breeding Base of Green Chemistry–Synthesis Technology, Zhejiang University of Technology, Hangzhou 310014, People’s Republic of China

## Abstract

The title compound, C_14_H_14_BrNO_3_, contains a bicyclic ring system with four chiral centers. The absolute structure was established by the Flack method.

## Related literature

For the asymmetric Diels-Alder reaction, which in principle allows the formation of four contiguous asymmetric centers, see: Anrendt *et al.* (2000 ▶); Northrup & MacMillan (2002 ▶); Xu *et al.* (2007 ▶); Xu *et al.* (2008 ▶).
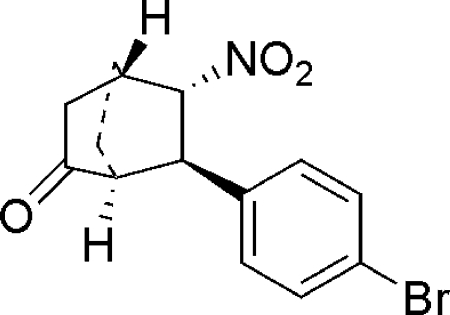


## Experimental

### 

#### Crystal data

C_14_H_14_BrNO_3_
*M* *_r_* = 324.17
Orthorhombic, 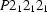

*a* = 6.4675 (8) Å
*b* = 10.0007 (13) Å
*c* = 20.108 (3) Å
*V* = 1300.6 (3) Å^3^
*Z* = 4
Mo *K*α radiation
μ = 3.16 mm^−1^
*T* = 293 (2) K
0.38 × 0.33 × 0.27 mm

#### Data collection

Bruker SMART APEX CCD area-detector diffractometer
Absorption correction: multi-scan (*SADABS*; Sheldrick, 1996 ▶) *T*_min_ = 0.758, *T*_max_ = 1.000 (expected range = 0.325–0.428)
7669 measured reflections
2823 independent reflections
2201 reflections with *I* > 2σ(*I*)
*R*_int_ = 0.057

#### Refinement

*R*[*F* ^2^ > 2σ(*F* ^2^)] = 0.038
*wR*(*F* ^2^) = 0.084
*S* = 0.90
2823 reflections
173 parameters
H-atom parameters constrained
Δρ_max_ = 0.63 e Å^−3^
Δρ_min_ = −0.53 e Å^−3^
Absolute structure: Flack (1983 ▶), 1161 Friedel pairs
Flack parameter: 0.024 (12)

### 

Data collection: *SMART* (Bruker, 2001 ▶); cell refinement: *SAINT* (Bruker, 2000 ▶); data reduction: *SAINT*; program(s) used to solve structure: *SHELXS97* (Sheldrick, 2008 ▶); program(s) used to refine structure: *SHELXL97* (Sheldrick, 2008 ▶); molecular graphics: *SHELXTL* (Sheldrick, 2008 ▶); software used to prepare material for publication: *SHELXTL*.

## Supplementary Material

Crystal structure: contains datablocks global, I. DOI: 10.1107/S1600536809001275/pv2129sup1.cif

Structure factors: contains datablocks I. DOI: 10.1107/S1600536809001275/pv2129Isup2.hkl

Additional supplementary materials:  crystallographic information; 3D view; checkCIF report

## Figures and Tables

**Fig. 1.**
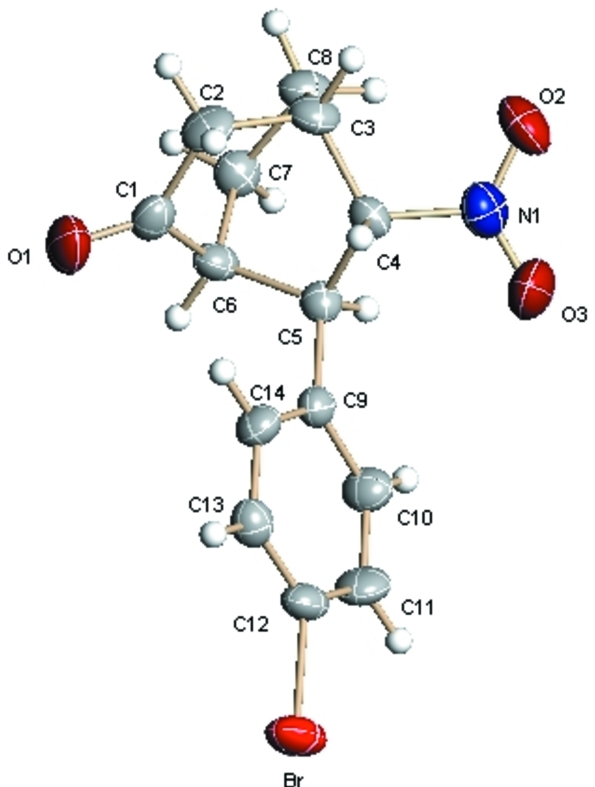
Molecular structure of the title compound with atomic labeling scheme; displacement ellipsoids are drawn at the 30% probability level.

**Fig. 2.**
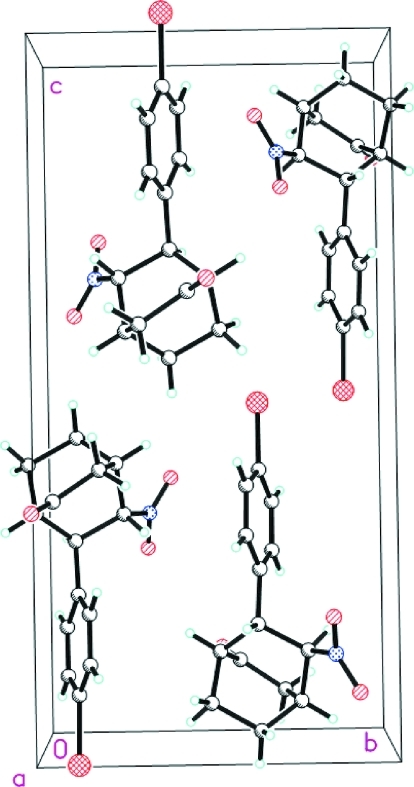
Unit cell packing of the title compound.

## supplementary crystallographic information

### Comment

There has been growing interest in the study of asymmetric Diels-Alder reaction because it allows in principle the formation of four contiguous asymmetric centers (Anrendt *et al.*, 2000; Northrup & MacMillan, 2002; Xu *et al.*, 2007, 2008). Consequently, we have synthesized a series of Diels-Alder products in our laboratory and the crystal structure and absolute configuration of one of these, the title compound, (I), is reported in this article.

In the title compound (Fig. 1), the nitryl and the 4-bromophenyl groups lie on different sides of the plane defined by C3/C4/C5 atoms with O2—N1—C4—C5 and N1—C4—C5—C9 torsion angles of 141.4 (4) and 92.5 (5)°, respectively. The C5—H5 bond and 4-bromophenyl group are almost coplanar with H5—C5—C9—C10 torsion angle of -2.3°. The structure is devoid of any classical hydrogen bonding and the molecules of (I) are separated by normal van der Waal's forces (Fig. 2).

### Experimental

A THF (1.0 ml) solution of *trans*-nitrostyrene (0.75 mmol) and cyclohex-2-enone (1.0 mmol) in the presence of (*S*)-1-methyl-2-(pyrrolidin-2-ylmethylthio)-1*H*-imidazole (0.15 mmol) as amine catalyst and benzoic acid (0.15 mmol) as additive at room temperature was subjected to vigorous stirring. After completion of the reaction, the mixture was washed with water (appoximately 300 ml) and extracted with ethyl acetate. The solvent was removed under reduced pressure and the residue was purified by silica gel column chromatography (eluent: petroleum ether-ethoxyethane). Single crystals were obtained by slow evaporation of an ethyl acetate solution.

### Refinement

H atoms were placed in calculated position with C—H = 0.98, 0.97 and 0.93 Å for methine, methylene and aryl H-atoms. All H atoms were included in the final cycles of refinement in riding mode, with *U*_iso_(H) = 1.2*U*_eq_ of the carrier atoms. An absolute structure was established using 1226 Friedel pairs and has been presented in this article.

### Crystal data

**Table:**

C_14_H_14_BrNO_3_	*F*(000) = 656
*M**_r_* = 324.17	*D*_x_ = 1.656 Mg m^−^^3^
Orthorhombic, *P*2_1_2_1_2_1_	Mo *K*α radiation, λ = 0.71073 Å
Hall symbol: P 2ac 2ab	Cell parameters from 2565 reflections
*a* = 6.4675 (8) Å	θ = 4.6–48.7°
*b* = 10.0007 (13) Å	µ = 3.16 mm^−^^1^
*c* = 20.108 (3) Å	*T* = 293 K
*V* = 1300.6 (3) Å^3^	Prismatic, colorless
*Z* = 4	0.38 × 0.33 × 0.27 mm

### Data collection

**Table:**

Bruker SMART APEX CCD area-detector diffractometer	2823 independent reflections
Radiation source: fine-focus sealed tube	2201 reflections with *I* > 2σ(*I*)
graphite	*R*_int_ = 0.057
φ and ω scans	θ_max_ = 27.0°, θ_min_ = 2.0°
Absorption correction: multi-scan (*SADABS*; Sheldrick, 1996)	*h* = −8→8
*T*_min_ = 0.758, *T*_max_ = 1.000	*k* = −12→12
7669 measured reflections	*l* = −25→18

### Refinement

**Table:**

Refinement on *F*^2^	Hydrogen site location: inferred from neighbouring sites
Least-squares matrix: full	H-atom parameters constrained
*R*[*F*^2^ > 2σ(*F*^2^)] = 0.038	*w* = 1/[σ^2^(*F*_o_^2^) + (0.043*P*)^2^] where *P* = (*F*_o_^2^ + 2*F*_c_^2^)/3
*wR*(*F*^2^) = 0.084	(Δ/σ)_max_ = 0.010
*S* = 0.90	Δρ_max_ = 0.63 e Å^−^^3^
2823 reflections	Δρ_min_ = −0.53 e Å^−^^3^
173 parameters	Extinction correction: *SHELXL97* (Sheldrick, 2008), Fc^*^=kFc[1+0.001xFc^2^λ^3^/sin(2θ)]^-1/4^
0 restraints	Extinction coefficient: 0.0251 (15)
Primary atom site location: structure-invariant direct methods	Absolute structure: Flack (1983), 1161 Friedel pairs
Secondary atom site location: difference Fourier map	Flack parameter: 0.024 (12)

### Special details

**Table:**

Geometry. All e.s.d.'s (except the e.s.d. in the dihedral angle between two l.s. planes) are estimated using the full covariance matrix. The cell e.s.d.'s are taken into account individually in the estimation of e.s.d.'s in distances, angles and torsion angles; correlations between e.s.d.'s in cell parameters are only used when they are defined by crystal symmetry. An approximate (isotropic) treatment of cell e.s.d.'s is used for estimating e.s.d.'s involving l.s. planes.
Refinement. Refinement of *F*^2^ against ALL reflections. The weighted *R*-factor *wR* and goodness of fit *S* are based on *F*^2^, conventional *R*-factors *R* are based on *F*, with *F* set to zero for negative *F*^2^. The threshold expression of *F*^2^ > σ(*F*^2^) is used only for calculating *R*-factors(gt) *etc*. and is not relevant to the choice of reflections for refinement. *R*-factors based on *F*^2^ are statistically about twice as large as those based on *F*, and *R*- factors based on ALL data will be even larger.

### Fractional atomic coordinates and isotropic or equivalent isotropic displacement parameters (Å^2^)

**Table:**

	*x*	*y*	*z*	*U*_iso_*/*U*_eq_	
Br	0.60615 (7)	0.37916 (4)	0.992203 (17)	0.05507 (17)	
N1	0.1712 (5)	0.1860 (3)	0.66087 (18)	0.0450 (8)	
O1	0.8682 (4)	0.4856 (3)	0.64840 (13)	0.0589 (8)	
O2	0.1115 (4)	0.1257 (3)	0.61231 (14)	0.0583 (7)	
O3	0.0748 (5)	0.1934 (4)	0.71214 (16)	0.0764 (10)	
C1	0.7048 (6)	0.4361 (4)	0.63535 (17)	0.0390 (9)	
C2	0.6837 (6)	0.3111 (4)	0.5941 (2)	0.0446 (10)	
H2A	0.7602	0.2389	0.6148	0.054*	
H2B	0.7415	0.3264	0.5502	0.054*	
C3	0.4587 (5)	0.2721 (4)	0.58773 (18)	0.0381 (9)	
H3	0.4457	0.1883	0.5628	0.046*	
C4	0.3809 (5)	0.2536 (3)	0.65847 (16)	0.0321 (7)	
H4	0.4797	0.1964	0.6821	0.038*	
C5	0.3716 (5)	0.3892 (3)	0.69495 (14)	0.0306 (7)	
H5	0.2273	0.4190	0.6935	0.037*	
C6	0.4991 (6)	0.4916 (4)	0.65427 (17)	0.0353 (8)	
H6	0.5158	0.5749	0.6793	0.042*	
C7	0.3820 (6)	0.5176 (3)	0.58930 (16)	0.0394 (8)	
H7A	0.4634	0.5755	0.5608	0.047*	
H7B	0.2523	0.5623	0.5989	0.047*	
C8	0.3387 (5)	0.3837 (4)	0.55301 (16)	0.0432 (9)	
H8A	0.1918	0.3642	0.5541	0.052*	
H8B	0.3815	0.3902	0.5069	0.052*	
C9	0.4318 (4)	0.3813 (4)	0.76760 (14)	0.0316 (7)	
C10	0.2904 (6)	0.4189 (3)	0.81598 (18)	0.0405 (9)	
H10	0.1590	0.4467	0.8033	0.049*	
C11	0.3406 (6)	0.4160 (4)	0.88210 (17)	0.0454 (10)	
H11	0.2429	0.4391	0.9140	0.054*	
C12	0.5348 (5)	0.3790 (4)	0.90080 (15)	0.0384 (8)	
C13	0.6804 (6)	0.3425 (3)	0.85488 (17)	0.0374 (9)	
H13	0.8123	0.3169	0.8681	0.045*	
C14	0.6269 (5)	0.3446 (3)	0.78835 (16)	0.0365 (8)	
H14	0.7252	0.3207	0.7567	0.044*	

### Atomic displacement parameters (Å^2^)

**Table:**

	*U*^11^	*U*^22^	*U*^33^	*U*^12^	*U*^13^	*U*^23^
Br	0.0759 (3)	0.0602 (3)	0.0292 (2)	−0.0056 (2)	−0.00768 (18)	−0.00532 (18)
N1	0.049 (2)	0.0307 (16)	0.055 (2)	−0.0033 (14)	−0.0048 (17)	0.0069 (15)
O1	0.0375 (17)	0.081 (2)	0.0584 (18)	−0.0188 (17)	−0.0023 (14)	0.0023 (15)
O2	0.0513 (15)	0.0495 (16)	0.0741 (18)	−0.0076 (18)	−0.0139 (15)	−0.0171 (16)
O3	0.076 (2)	0.090 (2)	0.062 (2)	−0.040 (2)	0.0159 (18)	0.0028 (18)
C1	0.038 (2)	0.048 (2)	0.031 (2)	−0.0038 (18)	0.0005 (16)	0.0104 (16)
C2	0.041 (2)	0.049 (2)	0.044 (2)	0.0109 (18)	0.0129 (17)	−0.0059 (18)
C3	0.049 (2)	0.033 (2)	0.0330 (19)	0.0027 (17)	−0.0010 (15)	−0.0093 (15)
C4	0.0306 (17)	0.0311 (18)	0.0344 (17)	−0.0006 (17)	−0.0064 (16)	−0.0025 (13)
C5	0.0308 (16)	0.0275 (16)	0.0335 (16)	0.0018 (18)	0.0013 (14)	−0.0029 (15)
C6	0.047 (2)	0.0310 (19)	0.0283 (19)	−0.0028 (18)	−0.0020 (15)	−0.0039 (15)
C7	0.043 (2)	0.0356 (19)	0.0392 (19)	0.0000 (18)	−0.0011 (19)	0.0051 (15)
C8	0.060 (2)	0.0377 (19)	0.0321 (17)	0.002 (2)	−0.0035 (15)	−0.0038 (17)
C9	0.0344 (18)	0.0287 (16)	0.0317 (16)	−0.0055 (18)	0.0025 (12)	−0.0006 (15)
C10	0.0364 (18)	0.047 (2)	0.038 (2)	0.0073 (17)	0.0027 (16)	−0.0044 (16)
C11	0.052 (3)	0.050 (3)	0.034 (2)	0.0054 (18)	0.0104 (17)	−0.0127 (16)
C12	0.053 (2)	0.0354 (19)	0.0268 (16)	−0.0065 (19)	0.0000 (14)	−0.0052 (18)
C13	0.0367 (18)	0.035 (2)	0.041 (2)	−0.0027 (15)	−0.0031 (15)	0.0042 (15)
C14	0.0351 (19)	0.042 (2)	0.0323 (17)	0.0020 (16)	0.0073 (16)	−0.0009 (14)

### Geometric parameters (Å, °)

**Table:**

Br—C12	1.895 (3)	C6—C7	1.532 (5)
N1—O3	1.207 (4)	C6—H6	0.9800
N1—O2	1.211 (4)	C7—C8	1.550 (5)
N1—C4	1.516 (4)	C7—H7A	0.9700
O1—C1	1.196 (4)	C7—H7B	0.9700
C1—C6	1.491 (5)	C8—H8A	0.9700
C1—C2	1.506 (5)	C8—H8B	0.9700
C2—C3	1.512 (5)	C9—C14	1.379 (5)
C2—H2A	0.9700	C9—C10	1.387 (4)
C2—H2B	0.9700	C10—C11	1.369 (5)
C3—C4	1.520 (5)	C10—H10	0.9300
C3—C8	1.529 (5)	C11—C12	1.362 (5)
C3—H3	0.9800	C11—H11	0.9300
C4—C5	1.543 (4)	C12—C13	1.368 (5)
C4—H4	0.9800	C13—C14	1.382 (5)
C5—C9	1.514 (4)	C13—H13	0.9300
C5—C6	1.549 (5)	C14—H14	0.9300
C5—H5	0.9800		
			
O3—N1—O2	123.7 (3)	C1—C6—H6	110.4
O3—N1—C4	117.5 (3)	C7—C6—H6	110.4
O2—N1—C4	118.8 (3)	C5—C6—H6	110.4
O1—C1—C6	125.3 (3)	C6—C7—C8	110.1 (3)
O1—C1—C2	123.0 (4)	C6—C7—H7A	109.6
C6—C1—C2	111.6 (3)	C8—C7—H7A	109.6
C1—C2—C3	110.4 (3)	C6—C7—H7B	109.6
C1—C2—H2A	109.6	C8—C7—H7B	109.6
C3—C2—H2A	109.6	H7A—C7—H7B	108.1
C1—C2—H2B	109.6	C3—C8—C7	108.9 (3)
C3—C2—H2B	109.6	C3—C8—H8A	109.9
H2A—C2—H2B	108.1	C7—C8—H8A	109.9
C2—C3—C4	105.7 (3)	C3—C8—H8B	109.9
C2—C3—C8	109.8 (3)	C7—C8—H8B	109.9
C4—C3—C8	110.4 (3)	H8A—C8—H8B	108.3
C2—C3—H3	110.3	C14—C9—C10	117.6 (3)
C4—C3—H3	110.3	C14—C9—C5	122.8 (3)
C8—C3—H3	110.3	C10—C9—C5	119.6 (3)
N1—C4—C3	112.4 (3)	C11—C10—C9	121.3 (3)
N1—C4—C5	110.0 (3)	C11—C10—H10	119.4
C3—C4—C5	110.5 (3)	C9—C10—H10	119.4
N1—C4—H4	107.9	C12—C11—C10	119.5 (3)
C3—C4—H4	107.9	C12—C11—H11	120.3
C5—C4—H4	107.9	C10—C11—H11	120.3
C9—C5—C4	113.7 (3)	C11—C12—C13	121.4 (3)
C9—C5—C6	114.1 (3)	C11—C12—Br	119.5 (3)
C4—C5—C6	108.0 (3)	C13—C12—Br	119.2 (3)
C9—C5—H5	106.9	C12—C13—C14	118.5 (3)
C4—C5—H5	106.9	C12—C13—H13	120.7
C6—C5—H5	106.9	C14—C13—H13	120.7
C1—C6—C7	106.7 (3)	C9—C14—C13	121.7 (3)
C1—C6—C5	111.3 (3)	C9—C14—H14	119.1
C7—C6—C5	107.4 (3)	C13—C14—H14	119.1
			
O1—C1—C2—C3	178.7 (4)	C9—C5—C6—C7	−163.8 (3)
C6—C1—C2—C3	−4.0 (4)	C4—C5—C6—C7	68.7 (3)
C1—C2—C3—C4	−58.0 (4)	C1—C6—C7—C8	65.1 (4)
C1—C2—C3—C8	61.1 (4)	C5—C6—C7—C8	−54.4 (4)
O3—N1—C4—C3	−163.6 (3)	C2—C3—C8—C7	−52.1 (4)
O2—N1—C4—C3	17.8 (4)	C4—C3—C8—C7	64.1 (4)
O3—N1—C4—C5	−40.0 (4)	C6—C7—C8—C3	−9.6 (4)
O2—N1—C4—C5	141.4 (3)	C4—C5—C9—C14	63.5 (4)
C2—C3—C4—N1	−167.8 (3)	C6—C5—C9—C14	−61.0 (4)
C8—C3—C4—N1	73.5 (3)	C4—C5—C9—C10	−120.1 (3)
C2—C3—C4—C5	68.9 (3)	C6—C5—C9—C10	115.4 (3)
C8—C3—C4—C5	−49.8 (4)	C14—C9—C10—C11	−1.9 (5)
N1—C4—C5—C9	92.5 (3)	C5—C9—C10—C11	−178.5 (3)
C3—C4—C5—C9	−142.8 (3)	C9—C10—C11—C12	1.8 (6)
N1—C4—C5—C6	−139.8 (3)	C10—C11—C12—C13	−1.0 (6)
C3—C4—C5—C6	−15.1 (4)	C10—C11—C12—Br	178.5 (3)
O1—C1—C6—C7	119.8 (4)	C11—C12—C13—C14	0.4 (6)
C2—C1—C6—C7	−57.4 (4)	Br—C12—C13—C14	−179.1 (3)
O1—C1—C6—C5	−123.4 (4)	C10—C9—C14—C13	1.2 (5)
C2—C1—C6—C5	59.5 (4)	C5—C9—C14—C13	177.7 (3)
C9—C5—C6—C1	79.8 (3)	C12—C13—C14—C9	−0.5 (5)
C4—C5—C6—C1	−47.7 (4)		

## References

[bb1] Anrendt, K. A., Borths, C. J. & MacMillan, D. W. C. (2000). *J. Am. Chem. Soc.* **122**, 4243–4244.

[bb2] Bruker (2000). *SAINT* Bruker AXS Inc., Madison, Wisconsin, USA.

[bb3] Bruker (2001). *SMART* Bruker AXS Inc., Madison, Wisconsin, USA.

[bb4] Flack, H. D. (1983). *Acta Cryst.* A**39**, 876–881.

[bb5] Northrup, A. B. & MacMillan, D. W. C. (2002). *J. Am. Chem. Soc.* **124**, 2548–2549.

[bb6] Sheldrick, G. M. (1996). *SADABS* University Of Göttingen, Germany.

[bb7] Sheldrick, G. M. (2008). *Acta Cryst.* A**64**, 112–122. 10.1107/S0108767307043930 18156677

[bb8] Xu, D. Q., Luo, S. P., Wang, Y. F., Xia, A. B., Yue, H. D., Wang, L. P. & Xu, Z. Y. (2007). *Chem. Commun.* pp. 4393–4395. 10.1039/b708525g 17957297

[bb9] Xu, D. Q., Wang, Y. F., Luo, S. P., Zhang, S., Zhong, A. G., Chen, H. & Xu, Z. Y. (2008). *Adv. Synth. Catal.* pp. 2610–2616.

